# Combined Maxacalcitol/Betamethasone Butyrate Propionate Ointment Possesses Long‐Term Sustained Effects of Inducing Regulatory T Cells and Downregulating the Th17 Response, Even After Discontinuation of Its Application in Imiquimod‐Induced Psoriasiform Dermatitis

**DOI:** 10.1111/1346-8138.70031

**Published:** 2025-10-25

**Authors:** Teruo Shimizu, Masahiro Kamata, Hideya Uratsuji, Yoshiki Okada, Ayu Watanabe, Azusa Hiura, Yayoi Tomura, Yayoi Tada

**Affiliations:** ^1^ Department of Dermatology Teikyo University School of Medicine Tokyo Japan; ^2^ Maruho Co., Ltd. Osaka Japan

**Keywords:** betamethasone butyrate propionate, interleukin‐10, interleukin‐17, maxacalcitol, psoriasis, regulatory T cells

## Abstract

We often experience sustained improvement, even after discontinuation of treatment in psoriasis lesions treated with topical vitamin D_3_ (VD_3_) or the combination of topical corticosteroids and VD_3_. However, the underlying mechanisms of these sustained effects remain unclear. We explored mechanisms for the sustained effects of maxacalcitol (MCT) and combined MCT/betamethasone butyrate propionate (BBP) ointments using a murine psoriasiform dermatitis model induced by imiquimod (IMQ). IMQ was applied once daily to the shaved backs of mice for 6 days to induce psoriasiform dermatitis. MCT, BBP, combined MCT/BBP ointment, or their vehicles were treated for 3 days prior to IMQ application. IMQ was reapplied after 1, 2, or 3 weeks from the first IMQ application to investigate the sustained effects of their ointments. In the first application of IMQ, the administration of MCT, BBP, or MCT/BBP ointment improved clinical and pathological manifestations and reduced Th17‐related cytokines. Treatment with MCT or MCT/BBP showed an increase in IL‐10 mRNA expression and a higher count of Foxp3^+^ cells within the skin, but not in those with BBP. The induction of IL‐10 by MCT and MCT/BBP persisted until reapplication of IMQ 2 weeks later, although their effects diminished 3 weeks later. The reduction in Th17‐related cytokines was maintained up to 3 weeks later in MCT/BBP, whereas it was not observed 2 weeks later in MCT. In conclusion, MCT and MCT/BBP showed long‐term effects by induction of regulatory T cells and IL‐10. Additionally, MCT/BBP downregulated Th17‐related cytokines, which could contribute to the sustained improvement after discontinuation observed in clinical practice.

**Abbreviations**
BBP: betamethasone butyrate propionate
IL: interleukin
IMQ: imiquimod
LN: lymph node
MCT: maxacalcitol
PCR: polymerase chain reaction
Tregs: regulatory T cells
VD_3_
: vitamin D_3_

## 1 Introduction

Psoriasis is a chronic skin disorder characterized by erythematous and scaly plaques. Recently, it has been considered a systemic inflammatory disease associated with arthritis and metabolic syndrome. The etiology of this disease reportedly includes genetic factors; immunological abnormalities including Th1/Th17, dendritic cells, neutrophils, macrophages, and B cells; and abnormal differentiation and proliferation of keratinocytes [1, 2, 3, 4, 5]. The interleukin (IL)‐17/IL‐23 cytokine pathway plays a significant role in the pathogenesis of the disease since the biologics targeting these cytokines are highly effective [6, 7, 8]. As an animal psoriasis model, Van der Fits et al. reported that daily application of imiquimod (IMQ), a toll‐like receptor (TLR) 7/8 agonist, to the skin of mice could induce psoriasis‐like dermatitis [9]. This mouse model exhibits clinical signs and histological features similar to those observed in human psoriasis, such as skin erythema, epidermal thickening, and scaling; the cytokine profile in the lesion is also similar. Therefore, it is widely used as a psoriasis model in many studies [10, 11, 12, 13, 14, 15, 16, 17, 18, 19].

At present, various treatments are available for psoriasis, including topical agents [20], phototherapy, oral medicines, and biological agents [8, 21]. Among topical therapies—including vitamin D_3_ (VD_3_) analogues, corticosteroids, and their combination—topical VD_3_ has been shown to not only inhibit the activation of T cells but also to restore the function of regulatory T cells (Tregs) and to promote their proliferation [22, 23]. IL‐10 possesses the immunomodulatory effect and is secreted by Tregs as well as dendritic cells, macrophages, and B cells [24]. A deficiency in IL‐10 levels within the skin and serum of psoriasis patients plays a critical role in the pathogenesis of the disease [25]. Moreover, the impaired function of Tregs is an essential factor in its pathogenesis [26]. We previously reported that the application of maxacalcitol (MCT), a VD_3_ analogue, resulted in an increase of Tregs and IL‐10 production and the suppression of Th17 responses, leading to improvement of psoriasis‐like dermatitis provoked by IMQ in mice, whereas topical corticosteroids enabled improvement of dermatitis through the suppression of Th17 responses without induction of Tregs [14]. As shown in our previous study [14], without IMQ application, treatment with MCT or BBP alone did not increase the number of Tregs, which remained few as in untreated mice.

In clinical practice, we frequently experience sustained improvement after discontinuation of application in lesions treated with topical VD_3_ or the combination of corticosteroid and VD_3_, while rapid recurrence is observed after discontinuation of application in lesions treated with topical corticosteroid [27, 28]. Although we hypothesize that Tregs induced by topical VD_3_ play an important role in exerting their sustained effects, it is poorly understood how long the immunosuppressive effect of peripheral Tregs is sustained.

In this study, using an IMQ‐induced psoriasiform dermatitis murine model, we investigated the sustained effects of immune cells (especially focusing on Tregs) after discontinuation of application in lesions treated with topical VD_3_ (MCT), corticosteroid (betamethasone butyrate propionate [BBP]), or the combination.

## 2 Materials and Methods

### 2.1 Animals

BALB/c mice were sourced from Sankyo Labo Service Corporation, Tokyo, Japan. The mice utilized for the experiment were females aged between 6 and 10 weeks and were kept in a specific pathogen‐free environment within the animal facility of Teikyo University School of Medicine. The study received approval from the Animal Research Committee of Teikyo University School of Medicine, under the approval numbers 19‐011, 19‐031.

### 2.2 Reagents

Twenty‐five μg/g maxacalcitol ointment (MCT, Oxarol; Maruho Co. Ltd., Osaka, Tokyo, Japan), 0.05% BBP ointment (Antebate; Torii Pharmaceutical Co. Ltd., Tokyo, Japan), and the combined MCT/BBP ointments (MCT/BBP, Marduox; Maruho Co. Ltd.) were all obtained from commercial sources. Vehicles of MCT and MCT/BBP were provided by Maruho Co. Ltd. For flow cytometric analysis, the following reagents were acquired: FITC‐conjugated anti‐mouse CD3, PerCP/Cy5.5‐conjugated anti‐mouse CD4, and Brilliant Violet 421‐conjugated anti‐mouse CD25, all of which were sourced from BioLegend, San Diego, CA. Additionally, PE‐conjugated anti‐mouse/rat Foxp3 was obtained from Thermo Fisher Scientific, Waltham, MA. For the immunostaining, goat anti‐mouse CD3ε (M‐20) was sourced from Santa Cruz Biotechnology, Santa Cruz, CA. Purified rat anti‐mouse/rat Foxp3 mAb was obtained from Thermo Fisher Scientific.

### 2.3 Induction of Psoriasiform Dermatitis by IMQ Application and Treatment of Ointments

**FIGURE 1:**
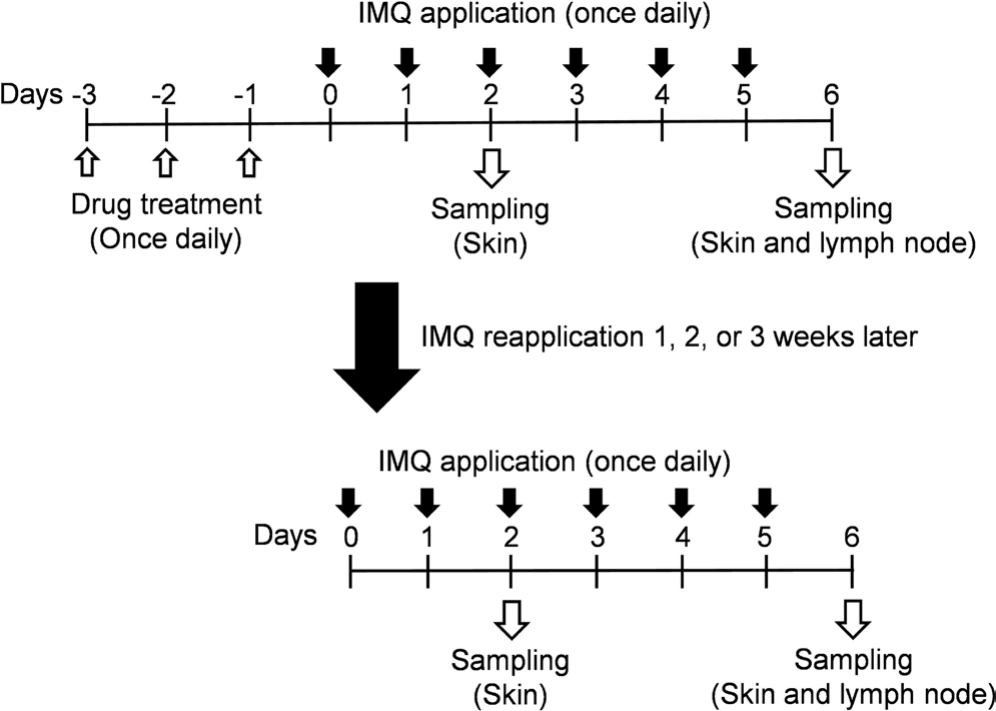
Experimental schedule. Psoriasiform dermatitis was provoked by imiquimod (IMQ) application once daily on the murine shaved back skin for 6 days. Maxacalcitol (MCT), betamethasone butyrate propionate (BBP), the combined MCT/BBP ointment, or their vehicles were treated for 3 days prior to IMQ application. IMQ was reapplied on mice after 1, 2, or 3 weeks from the first IMQ application to investigate the sustained effects of the ointments.

The experimental schedule for this study is summarized in Figure 1. Following the methodology of our previous experiments [16], we developed the IMQ‐induced psoriasiform dermatitis and treated each ointment. Briefly, MCT, BBP, MCT/BBP, vehicle of MCT, or MCT/BBP were administered to the shaved back skin once daily from day −3 to day −1. Then, 5% IMQ cream (Beselna Cream, Mochida Pharmaceuticals, Tokyo, Japan) was applied once daily from day 0 to 5. Furthermore, IMQ was reapplied on mice in the same methods after 1, 2, or 3 weeks from the first IMQ application. In each experiment of the first or the second IMQ application, the back skin samples of mice were collected on day 2 for quantitative real‐time polymerase chain reaction (PCR) analysis, and the back skin and inguinal lymph node (LN) samples were collected on day 6 for histological and flow cytometric analyses, respectively.

### 2.4 Histological Examination

The skin samples were prepared for histological examination and immunohistochemical analysis by embedding in Optimal Cutting Temperature Compound from Sakura Finetek (Tokyo, Japan) and then rapidly frozen in liquid nitrogen. For immunohistochemistry, these frozen skin samples were sectioned into 5‐μm‐thick slices, fixed with cold acetone, and subsequently washed with PBS. To minimize nonspecific antibody binding, samples were treated with a blocking solution and then incubated at 4°C overnight with either the primary antibodies specific for the targets or with isotype controls. Following this incubation, the slides were washed and then exposed to the corresponding secondary antibodies or reagents for 1 h. After a further wash, the slides were treated with an avidin‐biotin peroxidase complex developed using diaminobenzidine to visualize the antigen–antibody reactions, and counterstained with Mayer hematoxylin. The measurement of epidermal thickness was conducted on six randomly chosen fields per section at 400× magnification using Olympus cellSens Standard software (Olympus, Tokyo, Japan). The numbers of CD3‐ and Foxp3‐positive cells were quantified in five randomly selected fields per section at the same magnification.

### 2.5 Quantitative Real‐Time PCR

Total RNA was extracted from skin samples collected on day 2 from the backs of mice using RNeasy Fibrous Tissue Mini‐Kit (Qiagen, Valencia, CA). Subsequent cDNA synthesis was performed utilizing ReverTra Ace qPCR RT Master Mix from ToYoBo (Osaka, Japan). The quantification of gene expression in murine skin samples was carried out using the THUNDERBIRD Probe qPCR Mix from ToYoBo. Primers used for the experiments, for IL‐17F and IL‐22, were sourced from Thermo Fisher Scientific, while primers for IL‐17A, IL‐10, and GAPDH were obtained from Integrated DNA Technologies (San Diego, CA). All samples were analyzed with technical duplicates, with the expression of the GAPDH gene serving as an internal control. The relative expression levels of the target genes were calculated employing the 2^–ΔΔCT^ method.

### 2.6 Flow Cytometric Analysis

Inguinal LNs were harvested from mice on day 6 and minced through a 50 μm mesh to achieve single‐cell suspensions. These suspensions were then labeled with a fixable viability dye from BioLegend (San Diego, CA) and stained with cell surface antibodies targeting CD3, CD4, and CD25. Following surface staining, cells were fixed and permeabilized with Foxp3/Transcription Factor Staining Buffer Set from Thermo Fisher Scientific and subsequently stained with an antibody specific for Foxp3 for intracellular staining. The prepared cells were analyzed on a FACS Aria IIIμ flow cytometer, with data analysis conducted using FlowJo v10 software by Becton Dickinson (San Jose, CA).

### 2.7 Statistical Analysis

Data were obtained from three separate experiments and are expressed as mean ± standard error. For the purpose of conducting multiple comparisons across different groups, a one‐way analysis of variance (ANOVA) was employed, followed by Bonferroni's post‐test. Statistical significance was determined by *p* values less than 0.05 (*p < 0.05, **p < 0.01).

## 3 Results

### 3.1 Topical Treatment With MCT or MCT/BBP Ointment Reduced Psoriatic Skin Inflammation Induced by the First Application of IMQ

**FIGURE 2:**
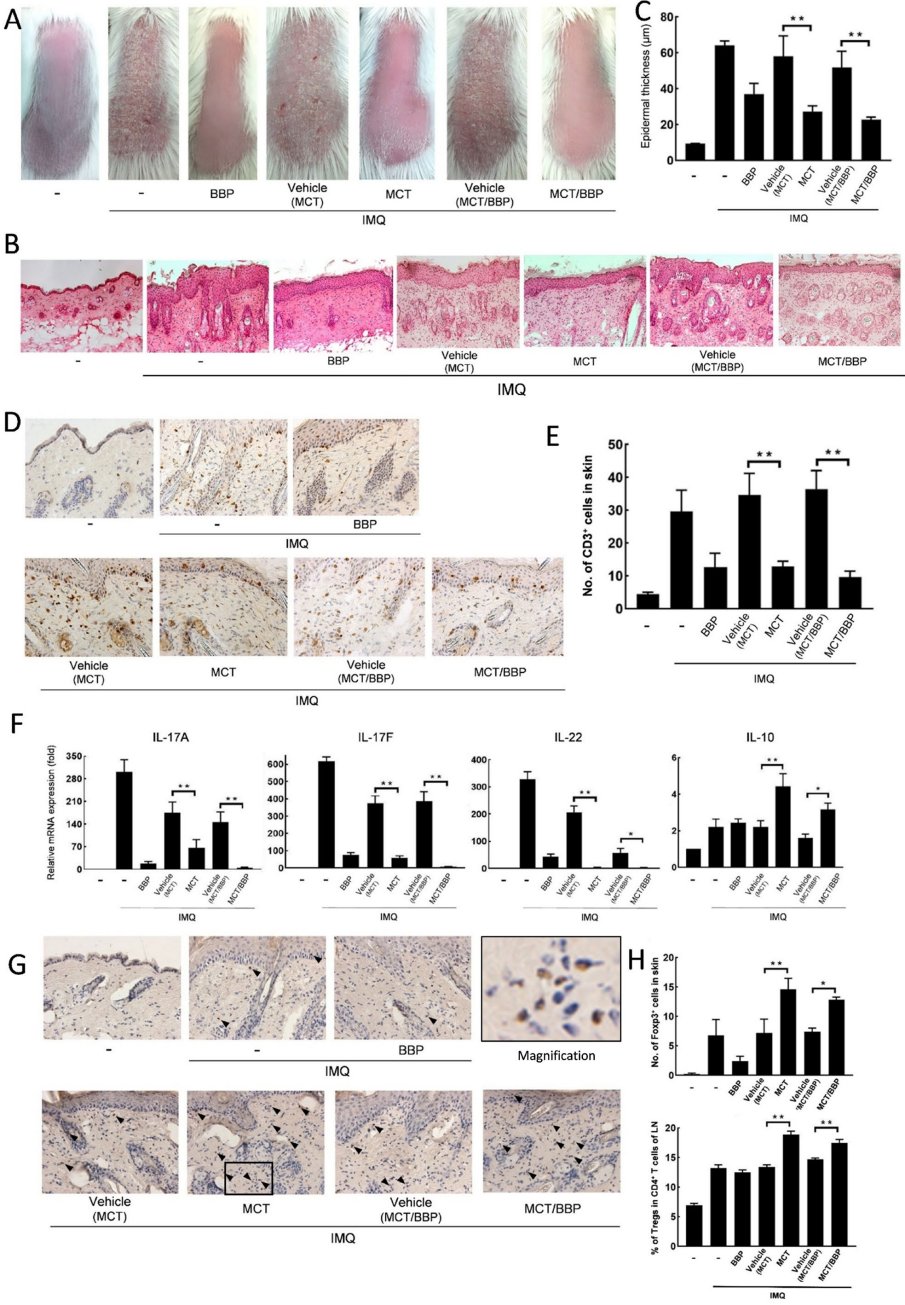
Topical treatment of MCT and MCT/BBP improves IMQ‐induced psoriasiform dermatitis through the inhibition of CD3^+^ cell infiltration and Th17‐related cytokine expression, and the induction of IL‐10 expression and Treg infiltration in the first IMQ application. IMQ was applied on the back skin of vehicle‐, MCT‐, BBP‐, or MCT/BBP‐treated BALB/c mice, as shown in the experimental schedule in Figure 1. (A, B) Representative clinical and histological photographs of the skin and (C) epidermal thickness on day 6. (D) Representative findings of the skin by immunohistochemical staining for CD3^+^ cells, and (E) the number of CD3^+^ cells on day 6. (F) mRNA expression of Th17‐related cytokines and IL‐10 in the skin on day 2. (G) Representative histological photographs of the skin by immunohistochemical staining for Foxp3^+^ cells (black arrowhead), and (H) the number of Foxp3^+^ cells in the skin and percentage of regulatory T cells (Tregs) in CD4^+^ T cells of lymph nodes (LN) on day 6. Enlarged images are provided in panel G to allow clearer recognition of Foxp3‐positive cells. Data are expressed as mean ± standard error (*n* = 6). One‐way ANOVA was employed, followed by Bonferroni's post‐test for multiple comparisons. Statistical significance was determined by *p* values less than 0.05 (*p < 0.05, **p < 0.01).

To investigate the effects of MCT, BBP, and combined MCT/BBP ointments on murine psoriasiform inflammation, they or their vehicles were treated for 3 days before the first IMQ application on the back skin of mice. These ointments were treated once daily for 3 days. Thereafter, IMQ was applied once daily for 6 days. As shown in Figure 2A, the application of IMQ provoked psoriasiform inflammation characterized by increased skin thickness, scaling, and erythema. On day 6, the topical treatment of either MCT, BBP, or MCT/BBP ointment apparently reduced the signs of psoriasiform dermatitis. Consistent with the observed clinical signs, histological examinations of skin samples from the IMQ‐applied mice and IMQ‐applied mice treated with vehicles showed epidermal thickening (acanthosis) and intense inflammatory cell infiltrates (Figure 2B). In contrast, their pathological findings were attenuated in the skin lesions obtained from the IMQ‐applied mice treated with MCT, BBP, or MCT/BBP (Figure 2B). Topical treatment of MCT or MCT/BBP ointment significantly reduced the epidermal thickness on day 6 compared with each vehicle (Figure 2C). Regarding inflammatory cell infiltrates, immunohistochemical staining revealed that MCT or MCT/BBP significantly suppressed infiltration of CD3^+^ cells into the skin of IMQ‐induced mice (Figure 2D,E). Moreover, MCT or MCT/BBP significantly reduced the mRNA expression levels of Th17‐related cytokines, such as IL‐17A, IL‐17F, and IL‐22, in the skin, compared with their vehicles on day 2 (Figure 2F). Whereas these inflammatory Th17‐related cytokines were suppressed by the application of MCT, BBP, or MCT/BBP, IL‐10 mRNA expression was significantly increased by MCT and MCT/BBP, but not BBP (Figure 2F). Since IL‐10 is known to be produced from Tregs [15, 24], we also examined the number of Tregs in skin and LN on day 6 using immunohistochemical staining and flow cytometric analysis, respectively. The number of Tregs and Foxp3^+^ cells in the skin was significantly increased in mice treated with MCT or MCT/BBP (Figure 2G,H). Similarly, the number of Tregs was significantly increased in LNs from mice treated with MCT or MCT/BBP (Figure 2H). These results indicate that MCT and MCT/BBP ointments exerted inhibitory effects on the psoriasiform inflammation induced by IMQ application through the induction of Tregs in the skin and LN, accompanied by IL‐10 production, in addition to the suppression of Th17 cytokines, such as IL‐17A, IL‐17F, and IL‐22.

### 3.2 Topical Treatment of MCT or MCT/BBP Ointment Sustained the Efficacy on Psoriatic Skin Inflammation Induced by the Second Application of IMQ

**FIGURE 3:**
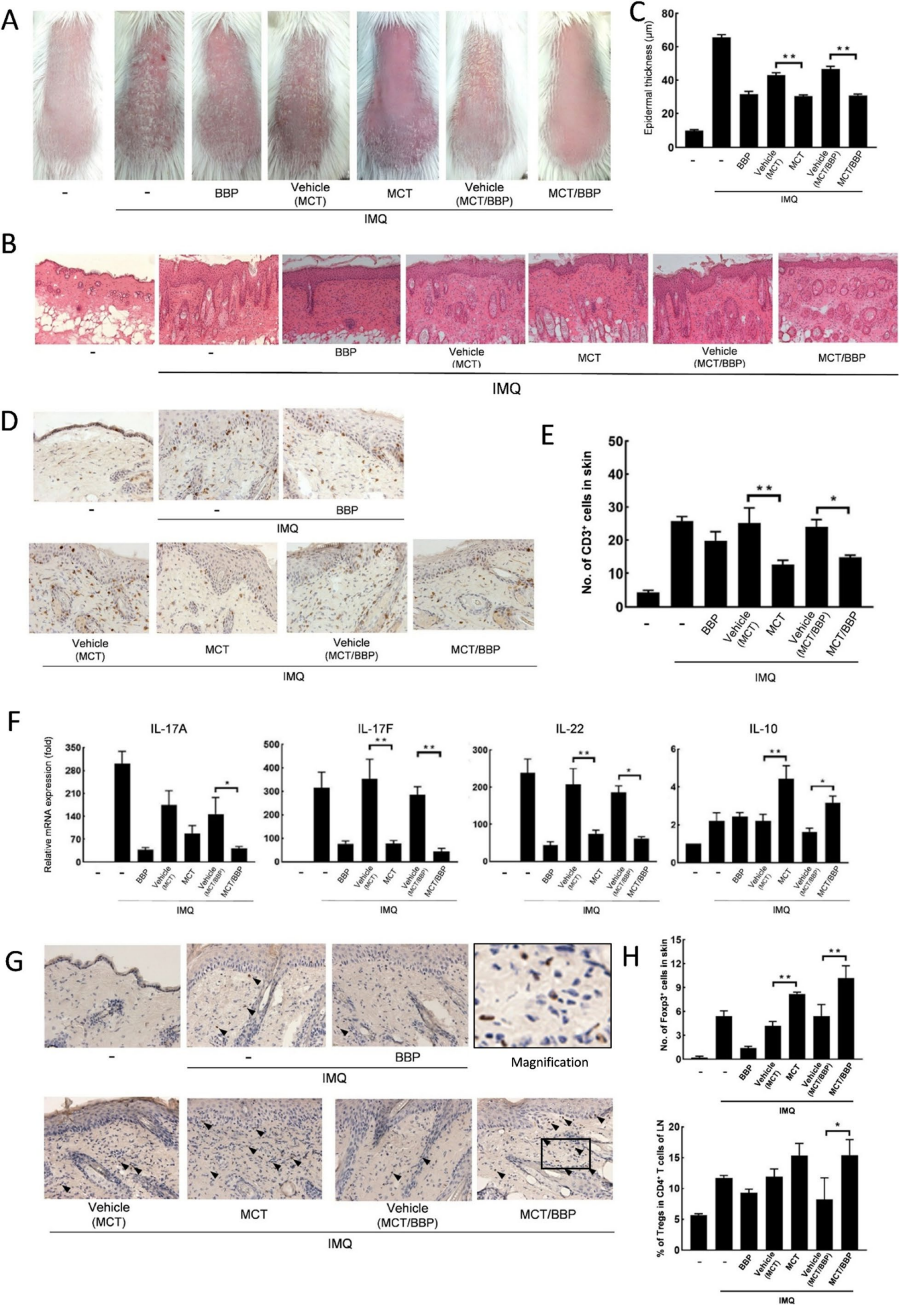
MCT and MCT/BBP maintain their effects of the inhibition of CD3^+^ cells infiltration and Th17‐related cytokine expression, and the induction of IL‐10 expression and Treg infiltration in the second IMQ application, 1 week later. (A, B) Representative clinical and histological photographs of the skin and (C) epidermal thickness on day 6. (D) Representative findings of the skin by immunohistochemical staining for CD3^+^ cells, and (E) the number of CD3^+^ cells on day 6. (F) mRNA expression of Th17‐related cytokines and IL‐10 in the skin on day 2. (G) Representative findings of the skin by immunohistochemical staining for Foxp3^+^ cells (black arrowhead), and (H) the number of Foxp3^+^ cells in skin and percentage of Tregs in CD4^+^ T cells of LN on day 6. Enlarged images are provided in panel G to allow clearer recognition of Foxp3‐positive cells. Data are expressed as mean ± standard error (*n* = 6). A one‐way ANOVA was employed, followed by Bonferroni's post‐test for multiple comparisons. Statistical significance was determined by *p* values less than 0.05 (*p < 0.05, **p < 0.01).

Next, to investigate how long the inhibitory effect of MCT, BBP, or MCT/BBP ointment was sustained on psoriasiform dermatitis induced by IMQ application, we reapplied IMQ once daily for 6 consecutive days after 1, 2, or 3 weeks from the first IMQ application (Figure 1) and evaluated it clinically and immunologically. The second IMQ application (1 week after completion of the first IMQ application) could induce psoriasiform inflammation clinically and histologically similar to the first IMQ application (Figure 3A,B). The topical treatment of MCT, BBP, or MCT/BBP ointment also showed similar effects on psoriasiform inflammation induced by the second IMQ application regarding the epidermal thickness and the number of CD3^+^ cells infiltrating into skin at day 6, and mRNA expression of Th17‐related cytokines in skin at day 2 (Figure 3C–F). Furthermore, MCT and MCT/BBP showed to maintain the effects of increased IL‐10 mRNA expression in skin at day 2 and the number of Tregs in the skin at day 6 compared with their vehicles, although there was no significant difference in Tregs of the LN of MCT‐treated mice (Figure 3F–H). These results indicate that the effects of MCT and MCT/BBP were sufficiently sustained, even 2 weeks after discontinuation of their treatment.

In the second IMQ application experiment (2 weeks after the first IMQ application), MCT and MCT/BBP ointment maintained similar effects regarding epidermal thickness, the number of CD3^+^ cells in the skin, and mRNA expression of IL‐10 (Figure S1). Although they showed a tendency to increase the number of Tregs in the skin and LN, no significant difference was observed compared to each vehicle (Figure S1). Regarding the expression of Th17‐related cytokines, MCT/BBP showed significant inhibitory effects compared to the vehicle, whereas MCT did not show significant inhibitory effects (Figure S1). These results indicate that the efficacy of MCT/BBP was well maintained, but the effect of MCT was slightly diminished.

**FIGURE 4:**
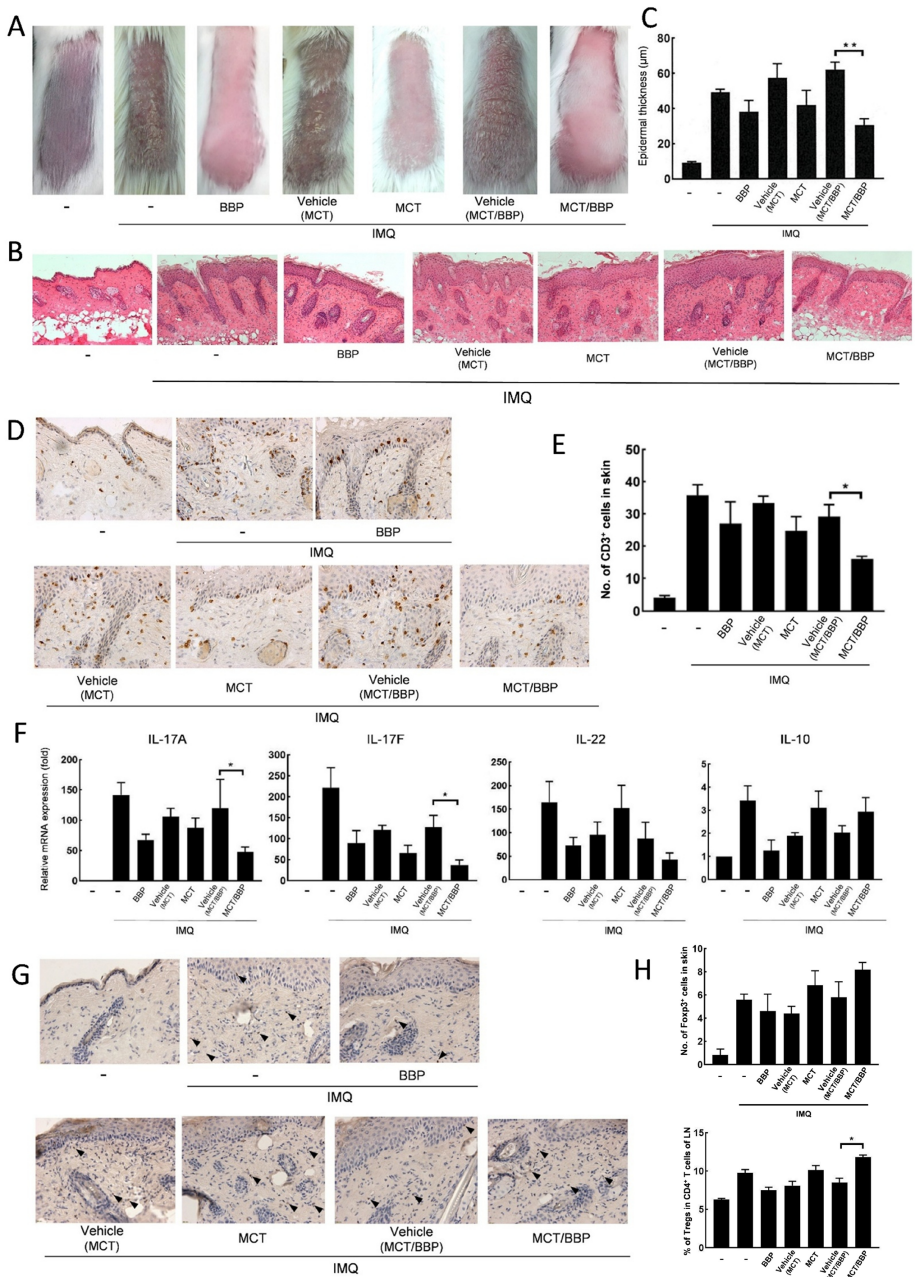
MCT/BBP maintains the effects of the inhibition of CD3^+^ cells infiltration and IL‐17A/F expression, and the induction of Tregs in LN even in the second IMQ application 3 weeks later. (A, B) Representative clinical and histological photographs of the skin and (C) epidermal thickness on day 6. (D) Representative histological findings of the skin by immunohistochemical staining for CD3^+^ cells, and (E) the number of CD3^+^ cells on day 6. (F) mRNA expression of Th17‐related cytokines and IL‐10 in the skin on day 2. (G) Representative findings of the skin by immunohistochemical staining for Foxp3^+^ cells (black arrowhead), and (H) the number of Foxp3^+^ cells in the skin and percentage of Tregs in CD4^+^ T cells of LN on day 6. Data are expressed as mean ± standard error (*n* = 6). One‐way ANOVA was employed, followed by Bonferroni's post‐test for multiple comparisons. Statistical significance was determined by *p* values less than 0.05 (*p < 0.05, **p < 0.01). Statistical analyses were performed by Tukey's post hoc test; no significant difference was observed between MCT and MCT/BBP at 3 weeks.

Finally, we examined the effect of these ointments on psoriasis‐like dermatitis induced by the second IMQ application 3 weeks after the first IMQ application. As shown in Figure 4, while their effects on epidermal thickness and the number of CD3^+^ cells were weakened in MCT and BBP, MCT/BBP was able to maintain significant inhibitory effects on them compared to the vehicle. Although the reduction of CD3^+^ cells appeared greater in the MCT/BBP group than in the MCT group at 3 weeks, Tukey's post hoc test revealed no statistically significant difference. Furthermore, MCT/BBP also sustained a significant increase in the number of Tregs in the skin. Accordingly, these results indicate that MCT/BBP, which combines the effects of MCT and BBP, could maintain its efficacy for a longer period.

## 4 Discussion

In this study, we demonstrated that treatment with MCT, BBP, or MCT/BBP ointment clinically and histologically improved psoriasis dermatitis in mice induced by the first IMQ application. As the mechanisms of their effects, MCT, BBP, or MCT/BBP showed reduction of Th17‐related cytokine expression and suppression of CD3^+^ cell infiltration in the skin, and MCT or MCT/BBP showed the immunoregulatory effects of inducing IL‐10 expression and Treg infiltration in addition to downregulating the Th17 response. Furthermore, in experiments when IMQ was reapplied after 1, 2, or 3 weeks from the completion of the first IMQ application to investigate the sustained effects of these ointments, our study revealed that MCT/BBP sustained these effects over a longer period compared to other ointments, although these effects were gradually weakened in a time‐dependent manner. Although immunofluorescent staining was not performed in this study, our previous report demonstrated Foxp3/IL‐10 double‐positive cells by immunofluorescence in IMQ‐induced dermatitis treated with maxacalcitol [14], supporting the current immunohistochemical findings.

Regarding the first IMQ application experiment, we previously reported that MCT, despite a slightly different timing of treatment, exerted its effects on the IMQ model through similar mechanisms as in this study [14], and its reproducibility was confirmed. In addition, although BBP was used as a corticosteroid in this study, we previously obtained similar results using betamethasone valerate (BV) as a corticosteroid, including the lack of induction effects on IL‐10 and Tregs [14]. Meanwhile, since MCT/BBP is used exclusively in Japan, there are few reports regarding MCT/BBP using the IMQ model. In our study, MCT/BBP showed stronger effects on the IMQ model by combining the effects of MCT and BBP without diminishing them. As a combination VD_3_ and corticosteroid treatment for psoriasis, combined calcipotriol (Cal) and betamethasone dipropionate (BD) ointment is also used clinically [29, 30]. We previously investigated the effects of the Cal/BD ointment on the IMQ model. In the previous study, Cal, BD, and Cal/BD ointments demonstrated efficacy in the first IMQ application experiment, similar to the findings of the present study using MCT, BBP, and MCT/BBP, although there were some differences, such as the lack of suppression of IL‐17A expression by Cal alone, and the absence of induced IL‐10 expression with Cal/BV [16]. Several factors could be considered as reasons for these differences. Although Cal is a VD_3_ analogue as MCT and BD is a corticosteroid as BBP, they are different substances, which possibly accounts for subtle differences in function and cutaneous absorption efficiency. Indeed, MCT reportedly exhibited stronger pharmacological effects compared to other VD_3_ analogs, including Cal [31, 32]. In this study, it was demonstrated that MCT/BBP combines the immunosuppressive and immunoregulatory effects of both MCT and BBP without any diminishment, and this aspect might be considered an advantage of MCT/BBP over Cal/BV. We speculate that the early suppression of psoriatic inflammation by BBP may prevent the establishment of resident memory T cells or epigenetic modifications, thereby allowing MCT to maximally induce Tregs.

Since Van der Fits et al. reported that applying IMQ to mice could induce psoriasiform dermatitis in 2009 [9], the IMQ model has been extensively used in numerous experiments [10, 11, 12, 13, 14, 15, 16, 17, 18, 19]. However, when IMQ was reapplied after a certain interval following IMQ application, it was not fully understood what symptoms would develop. Therefore, our results showed that the second IMQ application could induce psoriasiform dermatitis, similar to the first IMQ application. This model in our study could be considered useful for examining the long‐term sustained efficacy of various drugs. In the second IMQ application experiment, where IMQ was reapplied after 1 week, all drugs showed similar effects to those observed in the experiment of the first IMQ application. In the reapplication experiment 2 weeks later, BBP maintained its suppression of Th17‐related cytokines, but the overall immunosuppressive effects appeared to be weakened, considering the results in epidermal thickening and the number of CD3^+^ cells infiltrating into the skin. Similarly, while MCT maintained the effect of increased IL‐10 expression, its suppression of Th17‐related cytokines diminished. Meanwhile, the effects of MCT/BBP showed a tendency to sustain its effects compared to each monotherapy, although a reduction of some effects was observed. Finally, in the reapplication experiment 3 weeks later, the effects of both MCT and BBP were diminished, except for the suppression of Th17‐related cytokine in BBP. In contrast, the effects of MCT/BBP were relatively sustained compared to MCT and BBP. Although the detailed mechanism by which MCT/BBP sustained its effects for a longer period than MCT and BBP remains unclear, it is speculated that the additive or synergistic interaction between MCT and BBP may contribute to the long‐term sustained effects of MCT/BBP. Since the active ingredients contained in these ointments are thought to be eliminated from the body quickly, even within 1 week following treatment, physiological changes (within the skin and the rest of the body) caused by the ingredients could be responsible for long‐term maintenance effects. Further investigations are necessary to elucidate these mechanisms.

In conclusion, MCT showed long‐term effects of inducing Tregs and IL‐10 expression. In addition, MCT/BBP downregulated Th17‐related cytokines, which could contribute to sustained improvement even after discontinuation of the treatment observed in clinical practice.

## Ethics Statement

This study was approved by the Animal Research Committee of Teikyo University School of Medicine (19‐011, 19‐031).

## Conflicts of Interest

M.K. received honoraria for lectures from Maruho Co. Ltd. H.U. was an employee of Maruho Co. Ltd. Y.T. received honoraria for lectures and grants for research from Maruho Co. Ltd. Y.T. is an Editorial Board member of the *Journal of Dermatology* and a coauthor of this article. To minimize bias, she was excluded from all editorial decision‐making related to the acceptance of this article for publication.

## Supporting information


**Figure S1:** MCT/BBP maintains the effects of the inhibition of CD3^+^ cells infiltration and Th17‐related cytokine expression, and the induction of IL‐10 expression in the second IMQ application 2 weeks later. (A, B) Representative clinical and histological photographs of the skin and (C) epidermal thickness on day 6. (D) Representative findings of the skin by immunohistochemical staining for CD3^+^ cells, and (E) the number of CD3^+^ cells on day 6. (F) mRNA expression of Th17‐related cytokines and IL‐10 in the skin on day 2. (G) Representative findings of the skin by immunohistochemical staining for Foxp3^+^ cells (black arrowhead), and (H) the number of Foxp3^+^ cells in the skin and percentage of Tregs in CD4^+^ T cells of LN on day 6. Data are expressed as mean ± standard error (*n* = 6). One‐way analysis of variance (ANOVA) was employed, followed by Bonferroni's post‐test multiple comparisons. Statistical significance was determined by *p* values less than 0.05 (*p < 0.05, **p < 0.01).

## Data Availability

The data that support the findings of this study are available from the corresponding author upon reasonable request.
