# Supplementary figures and images for: Combined Maxacalcitol/Betamethasone Butyrate Propionate Ointment Possesses Long‐Term Sustained Effects of Inducing Regulatory T Cells and Downregulating the Th17 Response, Even After Discontinuation of Its Application in Imiquimod‐Induced Psoriasiform Dermatitis
Source: J Dermatol. 2025 Oct 25;53(1):25–34. doi: 10.1111/1346-8138.70031 (PMC12784800; doi:10.1111/1346-8138.70031)

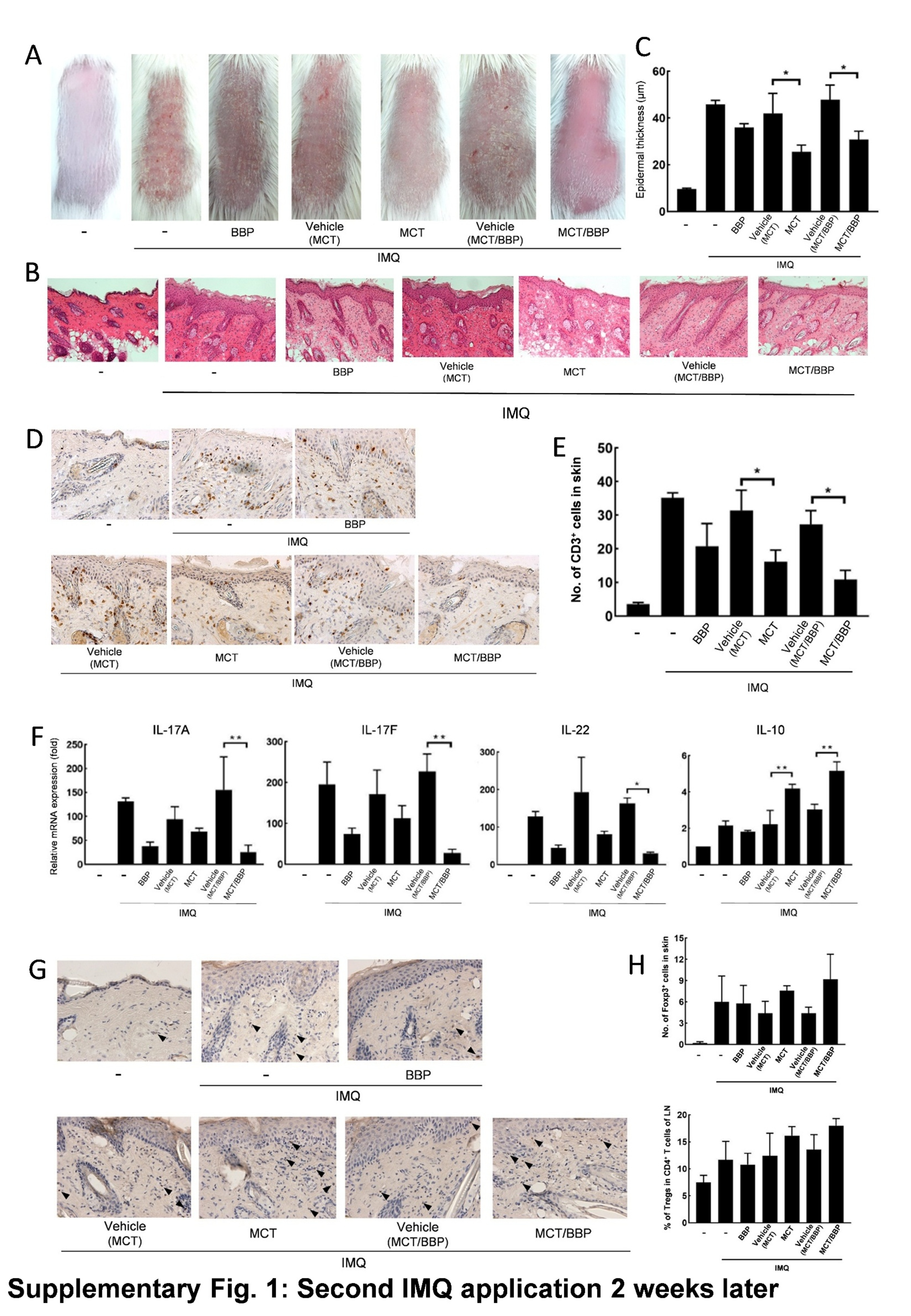

Supplement: Supplementary file 1 — Figure S1: MCT/BBP maintains the effects of the inhibition of CD3+ cells infiltration and Th17‐related cytokine expression, and the induction of IL‐10 expression in the second IMQ application 2 weeks later. (A, B) Representative clinical and histological photographs of the skin and (C) epidermal thickness on day 6. (D) Representative findings of the skin by immunohistochemical staining for CD3+ cells, and (E) the number of CD3+ cells on day 6. (F) mRNA expression of Th17‐related cytokines and IL‐10 in the skin on day 2. (G) Representative findings of the skin by immunohistochemical staining for Foxp3+ cells (black arrowhead), and (H) the number of Foxp3+ cells in the skin and percentage of Tregs in CD4+ T cells of LN on day 6. Data are expressed as mean ± standard error (n = 6). One‐way analysis of variance (ANOVA) was employed, followed by Bonferroni's post‐test multiple comparisons. Statistical significance was determined by p values less than 0.05 (*p < 0.05, **p < 0.01). [file JDE-53-25-s001.jpg]
